# Supplementary material for: Flanders Nursing Home (FLANH) project: Protocol of a multicenter longitudinal observational study on staffing, work environment, rationing of care, and resident and care worker outcomes
Source: PLoS One. 2023 Oct 26;18(10):e0293624. doi: 10.1371/journal.pone.0293624 (PMC10602233; doi:10.1371/journal.pone.0293624)
Supplement: Attachment — Submitted filename: Response to Reviewers.docx [file pone.0293624.s002.docx]

PLOS ONE

Editorial office

*September 22, 2023*

**Resubmission manuscript PONE-D-23-16826**

***Flanders Nursing Home (FLANH) project: Protocol of a multicenter longitudinal observational study on staffing, work environment, rationing of care, and resident and care worker outcomes***

Dear prof. dr. Jonas Preposi Cruz,

Thank you for the opportunity to revise and resubmit this manuscript for consideration for publication in PLOS ONE. We wish to thank the editor and reviewers for their constructive and insightful comments. We have revised our manuscript accordingly and believe this has greatly improved the manuscript.

Following this letter are the editor and reviewer comments with our responses in italics, including how and where the text was modified. Changes made in the manuscript are marked using track changes.

Yours sincerely,

On behalf of all authors,

Mieke Deschodt

**COMMENTS EDITOR**

1. Please ensure that your manuscript meets PLOS ONE's style requirements, including those for file naming.
2. One of the noted authors is a group or consortium. In addition to naming the author group, please list the individual authors and affiliations within this group in the acknowledgments section of your manuscript. Please also indicate clearly a lead author for this group along with a contact email address.

*We would like to refer to the Acknowledgments section (lines 310-318) where all members of the FLANH research consortium are listed. The authors of this manuscript are actually also part of the consortium and are hereby also added. The lead author of the consortium is prof. Mieke Deschodt, who is the corresponding author of this manuscript. Her contact email address is provided on the title page, and again added in the Acknowledgments section.*

3. Please review your reference list to ensure that it is complete and correct. If you have cited papers that have been retracted, please include the rationale for doing so in the manuscript text, or remove these references and replace them with relevant current references. Any changes to the reference list should be mentioned in the rebuttal letter that accompanies your revised manuscript. If you need to cite a retracted article, indicate the article’s retracted status in the References list and also include a citation and full reference for the retraction notice.

*No changes were made to the reference list.*

**COMMENTS REVIEWER #1**

1. In Data Sources, particularly, the survey development, it was mentioned in lines145 - 146 that the scales were translated if not available in Dutch. Provide the measured done or planned to ensure validity of the translated scales.

*Most of the scales and items were available in Dutch, but some indeed had to be translated. A bilingual researcher who was fluent in both English and Dutch performed a forward translation, which was then reviewed by a second bilingual researcher. In addition, as we describe in the manuscript, an expert panel review was performed were linguistic and conceptual equivalence was assessed. We also conducted a pre-test with end-users to identify any potential issues with comprehension or wording. All feedback was used to refine the translations further.*

*However, although we believe appropriate steps were taken to ensure the reliability of the translations, for pragmatic reasons no backward translation was performed. We acknowledge that this is a limitation and have therefore reformulated the sentence to make it clear that only forward translation was performed (lines 145-148): “Where necessary, adaptations were made for use in the nursing home setting (e.g. referring to ‘resident’ instead of ‘patient’) and for scales not available in Dutch, a forward translation was performed by a bilingual researcher.”*

2. In the Data Analysis, describe the analysis procedures and parameters in the interpretation of the included scales.

*Thank you for this suggestion. To provide more clarity, we have described the anchor of answer options and score interpretation for each included scale in Table 1. In the following results papers, we will of course go into more detail regarding scale calculation and transformation for statistical analysis.*

3. In the discussion section, strengths and limitations were explained. Limitations were backed up by planned measures to mitigate its effects. The authors might also want to consider adding the use of self-report questionnaires as a limitation and possible source of bias. Probable, the authors would like to add measures to address these limitations.

*Thank you for this suggestion. We acknowledge the importance of addressing this limitation and have added the following in the discussion section (lines 305-308): “Another potential risk to consider is response bias, as care workers may tend to give responses influenced by social desirability. To mitigate this, we will emphasize that all surveys will be sent directly to the research team and that confidentiality will be guaranteed.”*

**COMMENTS REVIEWER #2**

1. In line 172 explain what is understood by "productive hours"

*Thank you for this comment. We have added the following sentence to clarify this (lines 173-174): “Productive hours are the actual worked hours and do not include paid or scheduled hours for vacation, sick leave, or education.”*

2. Section from 186-200: are the quality indicators you use from the Flemish Institute for Quality of Care retrievable from the BelRAI database? If not, think about to give the section another title.

*Currently, the Flemish Institute for Quality of Care gathers the quality indicators at the nursing home level through self-reporting by nursing home management and not through the BelRAI. However, as part of the BelRAI assessment, these quality indicators and many more outcomes are now also being collected at the resident level. Although the Flemish Institute for Quality of Care is planning to switch to the BelRAI in the future, we already decided to retrieve these outcomes from the BelRAI database.*

*So to answer the question, all resident outcomes we will use will be retrieved from the BelRAI database and will not be obtained from the Flemish Institute for Quality of Care. We agree that the text reads ambiguously and have therefore removed the reference to the Flemish Institute for Quality of Care from the paragraph (lines 195-198).*
